# Quality of life among patients with age-related severe macular degeneration assessed using the NEI-VFQ, HADS-A, HADS-D and SF-36 tests. A cross-sectional study

**DOI:** 10.1590/1516-3180.2018.0195071218

**Published:** 2019-05-08

**Authors:** Sibel Inan, Ersan Cetinkaya, Resat Duman, Ismet Dogan, Umit Übeyt Inan

**Affiliations:** I MD. Assistant Professor, Department of Ophthalmology, Afyon Kocatepe Üniversitesi (AKÜ) Tıp Fakültesi, Afyonkarahisar, Turkey.; II MD. Ophthalmologist, Department of Ophthalmology, Manisa Devlet Hastanesi, Manisa, Turkey.; III MD. Assistant Professor, Department of Ophthalmology, Afyon Kocatepe Üniversitesi (AKÜ) Tıp Fakültesi, Afyonkarahisar, Turkey.; IV PhD. Professor,Department of Clinic Biostatistics, Afyon Kocatepe Üniversitesi (AKÜ) Tıp Fakültesi, Afyonkarahisar, Turkey.; V MD. Professor, Department of Ophthalmology, PARKHAYAT Hastanesi, Afyonkarahisar, Turkey.

**Keywords:** Quality of life, Macular degeneration, VFQ-25, HAD-A, SF-36

## Abstract

**BACKGROUND:**

Exudative age-related macular degeneration (e-AMD) may cause severe central vision loss. Patients with e-AMD can experience difficulties in daily basic activities and suffer from psychological problems. Our aim was to assess quality of life (QoL) and anxiety and depression status among patients with e-AMD.

**DESIGN AND SETTING:**

Cross-sectional study in a state university.

**METHODS:**

We included 200 e-AMD patients and 120 age and gender-matched controls. We assessed QoL using the National Eye Institute Visual Functioning Questionnaire-25 (NEI-VFQ-25) and the Short Form (SF)-36 test; and anxiety and depression status using the Hospital Anxiety Depression Scales A and D (HADS-A and HADS-D).

**RESULTS:**

The mean ages in the e-AMD and control groups were 68.40 ± 9.8 and 66.31 ± 8.98, respectively. Visual acuity among e-AMD patients was 0.37 ± 0.31 and 0.39 ± 0.32 in the right and left eyes, respectively. The e-AMD patients performed significantly worse than the controls in NEI-VFQ-25 (P < 0.05 for all items). The proportions of e-AMD patients scoring higher than the cutoffs in HADS-A and HADS-D were significantly higher than among the controls (41.5% versus 12.5% and 63.5% versus 27.5%; P < 0.001). The e-AMD patients had significantly lower mean scores than the controls for each of the SF-36 QoL items (P < 0.001). The NEI-VFQ-25 scores were significantly lower among patients with bilateral e-AMD than among those with unilateral disease (P < 0.05 for all). The HADS scores were positively correlated with duration of e-AMD and patient age, but negatively with vision levels (P < 0.05 for all items).

**CONCLUSION:**

The e-AMD patients had higher depression and anxiety scores and lower QoL scores.

## INTRODUCTION

Age-related macular degeneration (AMD) is a neurodegenerative disease of the retina characterized by loss of central vision in old age. In particular, wet-type or exudative AMD, which is characterized by choroidal neovascularization, may cause severe loss of vision in these patients. AMD is the leading cause of central blindness among patients aged over 65 years in developed countries. The prevalence of AMD among individuals aged between 65 and 75 years is 10% and it is 25% among those aged 75 years and over. Patients with AMD may face difficulties in relation to many of the basic activities of daily living, such as writing, housework, self-care, driving and shopping. They may also suffer emotional distress and depression, and have a reduced quality of life (QoL).^1^ Furthermore, loss of vision increases the risk of falling and fall-related injuries.^2^ In all of its aspects, AMD is recognized as an important public health problem.

Although the effects of exudative AMD (e-AMD) on quality of life, depression and physical and mental health have been studied more extensively in well-developed countries, data is relatively lacking from developing or underdeveloped countries. Moreover, differences in its effects may be seen between communities. Different social traits, belief sets and cultural characteristics in different communities may lead patients to be influenced psychologically in different manners from the same disease.

The hospital anxiety and depression scale (HADS) was designed to measure the risks of anxiety and depression and their levels. Thetest includes two subscales: HADS-A, which assesses anxiety, and HADS-D, which assesses depression, both including seven items. The cutoff points for HADS-A and HADS-D are ten and seven, respectively. Higher scores indicate a greater likelihood of anxiety and depression. The validity and reliability of HADS in the Turkish population were assessed by Aydemir etal.^3^

The National Eye Institute Visual Functioning Questionnaire (NEI-VFQ-25) scale was developed to determine QoL among patients with chronic blindness. The scale provides an evaluation of the impact of visual impairment on the emotional wellbeing, social relationships and daily activities of patients with chronic blindness. Its validity and reliability in the Turkish population were assessed by Toprak etal.^4^

Several other scales have been developed to assess physical and mental health.^5^ The Short Form 36 (SF-36) test is a general QoL scale assessing the physical and emotional health of patients. It consists of a total of 36 questions, classified in eight domains: physical functioning, role-physical, bodily pain, general health, social functioning, role-emotional, mental health and vitality. TheSF-36 scale is not specific to any disease, age or treatment group and represents measurements of general health. Because of its ease of use, the SF-36 test is particularly ideal for older patients. Its validity and reliability in the Turkish population were assessed by Kocyigit etal.^6^

## OBJECTIVE

In the current study, we aimed to evaluate the impact of e-AMD on the QoL of patients, using the HADS-A, HADS-D, NEI-VFQ-25 and SF-36 questionnaires.

## METHODS

This study was undertaken between February 2015 and May 2015. A cohort of consecutive patients with e-AMD was recruited from the medical retina department. Age and gender-matched controls with simple refractive errors were recruited from the eye polyclinic. We followed the principles of the Declaration of Helsinki; we obtained ethical approval from the Afyonkarahisar Clinical Research Ethics Board (approval number: 61; date of approval: January 29, 2015); and we obtained written informed consent from all of the patients and controls.

The patients with severe AMD were aged over 50 years, had exudative-type AMD and were already receiving anti-vascular endothelial growth factor (anti-VEGF) treatment on an as-needed basis to treat e-AMD. All of the patients in the study group had e-AMD in at least one eye, with Snellen visual acuity of 0.5 or less. ExudativeAMD was defined and diagnosed as occurrences of intraretinal and subretinal fluid caused by choroidal neovascularization. It was accompanied by drusen and retinal pigment epithelial detachments, which were demonstrated by means of fundus fluorescein angiography and spectral domain optical coherence tomography (Heidelberg Spectralis OCT and HRA, Heidelberg, Germany).

Patients with comorbid disorders were excluded from the study. In addition, patients with glaucoma, optic neuropathy, diabetic retinopathy, uveitis, amblyopia, degenerative myopia or cataracts were also excluded.

We recorded the age, education, gender, marital status, income levels and duration of the disease of all of the patients. We performed three tests on both the patients and the controls: the NEI-VFQ-25 test, the HADS test and the SF-36 questionnaire*.* Sociodemographic factors were also compared between the two groups.

The NEI-VFQ-25 test was developed to determine QoL among patients with chronic blindness. This test includes a total of 25questions that assess general health, general vision, vision-specific mental health, vision-specific social functioning, vision-specific dependency, ocular pain, near and distant activities, role limitations, color vision and peripheral vision.

The HADS test consists of a fourteen-item scale that was developed to detect states of anxiety and depression in a hospital setting.^7^ It has two subscales: HADS-A for assessing anxiety and HADS-D for assessing depression. Both scales include seven items that are scored from 0 to 3. Questions 1, 3, 5, 6, 8, 10, 11 and 13 are scored as 3, 2, 1 or 0; and questions 2, 4, 7, 9, 12 and 14 are scored as 0, 1, 2 or 3. While questions 1, 3, 5, 7, 9, 11 and 13 relate to anxiety, questions 2, 4, 6, 8, 10, 12 and 14 relate to depression. The total score ranges from 0 to 21 for both of the scales. The cutoff points for HADS-A and HADS-D are ten and seven, respectively. Total scores greater than these cutoffs indicate the existence of a risk of depression or anxiety.

The SF-36 QoL test includes a total of 36 self-assessment questions that are classified into eight domains: physical functioning with ten questions, role-physical with four, bodily pain with two, general health with five, social functioning with two, role-emotional with three, mental health with five and vitality with four. The reference period for all of the questions was the last four weeks prior to the interview.

The NEI-VFQ-25 test, the HADS test and the SF-36 questionnaire were applied to all the patients by one of the authors (SI).

We used the Statistical Package for the Social Sciences (SPSS) software, version 15.0, to perform the statistical analyses. We used the Kolmogorov-Smirnov test to assess the normality of distribution. We used the Pearson chi-square test to analyze any relationship between two categories of data. We used the Analysis of Variance test to analyze data with normal distribution and homogeneous variance. For continuous variables, we used the Student t test, Mann-Whitney U test and the Tukey HDS test. Existence of statistical significance was established at a p-value of < 0.05, and the confidence interval was taken to be 95%.

## RESULTS

A total of 200 patients and 120 controls were enrolled in the study. The mean ages in the e-AMD and control groups were 68.40 ± 9.8 and 66.31 ± 8.98 years, respectively (P = 0.06). Themean binocular visual acuity (according to a Snellen chart line) was 0.52±0.31 in the e-AMD group and 0.96 ± 0.04 in thecontrol group (P = 0.00). The visual acuity among patients in the e-AMD group was 0.37 ± 0.31 in the right eye and 0.39 ± 0.32 in the left eye (P = 0.80). There was no significant difference regarding smoking, education, sex or income levels between the control and patient groups (Table 1).

**Table 1.** Demographic characteristics of the patient and control groups

	e-AMD patients (n = 200)	Controls (n = 120)	P
Age			
Mean in years	68.40 ± 9.80	66.31 ± 8.98	0.06
Under 60 years	37	31	0.12
60 years and over	163	89	
Gender			
Female	111	61	0.24
Male	89	59	
BCVA (mean, Snellen chart line)			
Binocular	0.50 ± 0.30	0.98 ± 0.04	< 0.001
Right eyes with e-AMD	0.36 ± 0.30	0.97 ± 0.05	< 0.001
Left eyes with e-AMD	0.37 ± 0.30	0.95 ± 0.03	< 0.001
Duration of e-AMD (months)			
Right eyes	18.17 ± 20.02	-	-
Left eyes	18.74 ± 19.91	-	-
Laterality of e-AMD (n)			
Unilateral eyes	38 RE/24 LE	-	-
Bilateral eyes	138	-	-
Education			
Illiterate	22	17	0.930^a^
Literate	25	15	
Elementary	127	72	
High school	19	11	
University	7	5	
Income status			
Revenue smaller than expenditure	66	38	0.429^a^
Revenue equal to expenditure	108	60	
Revenue greater than expenditure	26	22	
Job			
Housewife	91	52	0.044^a^
Retired	72	31	
Farmer	20	12	
Public sector	7	12	
Private sector	6	8	
Not working	4	5	
Smoking			
Smoker*	39	26	0.641^a^
Non-smoker	161	94	

e-AMD = exudative age-related macular degeneration; BCVA = best corrected visual acuity; ^a^chi-square test. *Participants were considered to be smokers if they had smoked at least 100 cigarettes during their lifetimes and if they reported, at the time of the interview, that they smoked every day or on some days.

The patients in the e-AMD group performed significantly worse than the controls in all of the items of the NEI-VFQ-25 test (general health, general vision, vision-specific mental health, vision-specific social functioning, vision-specific dependency, ocular pain, near and distant activities, driving, color vision and peripheral vision) (P < 0.05 for all items). We were unable to compare driving performance. The driving test was not applicable tothe patients with e-AMD, because many of them were not drivers even before the onset of the disease. The NEI-VFQ-25 scores for general vision, near vision, peripheral vision, color vision, dependency and ocular pain were significantly lower among the patients with bilateral e-AMD than among those who had unilateral disease (Table 2).

**Table 2.** Differences in subscale scores in the National Eye Institute Visual Functioning Questionnaire-25 (NEI-VFQ-25), between the exudative age-related macular degeneration (e-AMD) group and control group and between the unilateral and bilateral exudative AMD subgroups

Subscales	NEI-VFQ-25 scores					P^b^
	All e-AMD patients (n = 200)	e-AMD patients		P^a^	Controls (n = 120)	
		Unilateral e-AMD (n = 62)	Bilateral e-AMD (n = 138)			
General health	35.8 ± 23.8	38.7 ± 22.9	34.4 ± 24.2	0.28	65.5 ± 14.1	< 0.001
General vision	42.5 ± 17.9	45.6 ± 16.0	41.0 ± 18.6	0.04	81.6 ± 9.7	< 0.001
Mental health	43.6 ± 23.2	47.2 ± 21.5	41.9 ± 23.8	0.12	95.3 ± 4.4	< 0.001
Ocular pain	49.8 ± 26.4	54.8 ± 23.8	47.5 ± 27.3	0.05	88.5 ± 11.3	< 0.001
Near vision	48.0 ± 26.4	54.1 ± 24.4	45.4 ± 26.9	0.03	92.9 ± 7.7	< 0.001
Distance vision	50.1 ± 24.4	56.9 ± 22.2	50.5 ± 25.2	0.17	92.3 ± 6.5	< 0.001
Social functioning	62.6 ± 29.4	68.9 ± 26.9	61.2 ± 30.3	0.13	98.3 ± 5.0	< 0.001
Role difficulties	61.0 ± 23.9	44.3 ± 22.9	40.2 ± 24.2	0.24	97.1 ± 7.7	< 0.001
Dependency	48.8 ± 25.1	54.7 ± 23.2	46.1 ± 25.5	0.02	98.0 ± 4.5	< 0.001
Color vision	68.4 ± 30.5	75.0 ± 28.0	65.4 ± 31.2	0.04	97.5 ± 7.5	< 0.001
Peripheral vision	53.9 ± 27.9	60.8 ± 23.3	50.7 ± 29.3	0.02	93.2 ± 11.1	< 0.001

P^a^ = comparison of unilateral and bilateral cases; P^b^ = comparison of e-AMD and control groups.

Based on binocular vision levels, the level of vision showed a positive correlation with the test scores in both groups. Similarly, as the level of vision decreased, so did the test scores. The scores were inversely and significantly correlated with the age and duration of the illness. The scores for emotional role difficulty, mental health, social functioning and pain on the SF-36 scale were negatively correlated with the duration of e-AMD and patient age, but were positively correlated with vision levels. The scores for HADSwere positively correlated with the duration of e-AMD and patient age, but were negatively correlated with vision levels. The correlations of scores for the subscales of NEI-VFQ-25, SF-36, HADS-A and HADS-D with the duration of e-AMD, the best corrected visual acuity (BCVA) in the better-seeing eye and the patients’ age are given in Table 3.

**Table 3.** Correlation of subscale scores in the National Eye Institute Visual Functioning Questionnaire-25 (NEI-VFQ-25), Short Form-36 (SF-36) and Hospital Anxiety Depression Scales A and B (HADS-A and HADS-D) tests with the duration of exudative age-related macular degeneration (e-AMD), best corrected visual acuity (BCVA) in the better-seeing eye and age

Scores	Significantly correlated parameters		
	Duration of e-AMD (n = 200)	BCVA in better-seeing eye (n = 200)	Age (n = 200)
Subscales of NEI-VFQ-25			
General health	P = 0.031; r = -0.16	P < 0.001; r = 0.46	P < 0.001; r = -0.30
General vision	P < 0.001; r = -0.25	P < 0.001; r = 0.58	P < 0.001; r = -0.27
Mental health	P < 0.001; r = -0.32	P < 0.001; r = 0.61	P < 0.001; r = -0.29
Ocular pain	P < 0.001; r = -0.32	P < 0.001; r = 0.52	P = 0.001; r = -0.19
Near vision	P = 0.001; r = -0.24	P < 0.001; r = 0.52	P < 0.001; r = -0.31
Distance vision	P = 0.068; r = -0.14	P < 0.001; r = 0.45	P < 0.001; r = -0.33
Social functioning	P = 0.002; r = -0.23	P < 0.001; r = 0.48	P < 0.001; r = -0.25
Role difficulties	P < 0.001; r = -0.25	P < 0.001; r = 0.61	P < 0.001; r = -0.23
Dependency	P < 0.001; r = -0.28	P < 0.001; r = 0.59	P < 0.001; r = -0.27
Color vision	P < 0.001; r = -0.30	P < 0.001; r = 0.42	P < 0.001; r = -0.20
Peripheral vision	P = 0.002; r = -0.21	P < 0.001; r = 0.51	P < 0.001; r = -0.22
Subscales of SF-36			
Physical functioning	P = 0.061; r = -0.13	P < 0.001; r = 0.25	P < 0.001; r = -0.36
Role limitation due to physical health	P = 0.067; r = -0.13	P < 0.001; r = 0.29	P < 0.001; r = -0.24
Role limitation due to emotional problems	P = 0.005; r = -0.20	P < 0.001; r = 0.27	P < 0.001; r = -0.22
Energy/fatigue	P = 0.231; r = -0.08	P < 0.001; r = 0.26	P = 0.001; r = -0.18
Emotional wellbeing	P = 0.033; r = -0.15	P < 0.001; r = 0.21	P = 0.002; r = -0.18
Social functioning	P = 0.002; r = -0.22	P < 0.001; r = 0.24	P < 0.001; r = -0.24
Pain	P < 0.001; r = -0.31	P < 0.001; r = 0.29	P < 0.001; r = -0.24
General health	P = 0.051; r = -0.14	P < 0.001; r = 0.29	P < 0.001; r = -0.28
HADS scores			
HADS-A score	P = 0.131; r = 0.17	P < 0.001; r = -0.38	P = 0.001; r = 0.18
HADS-D score	P = 0.005; r = 0.19	P < 0.001; r = -0.32	P = 0.002; r = 0.17

HADS-A and HADS-D scores were compared with visual acuity, laterality of disease and demographic characteristics among the patients with exudative AMD. This is shown in Table 4.

**Table 4.** HADS-A and HADS-D scores in comparison with demographic characteristics among patients with exudative age-related macular degeneration (e-AMD)

Groups		Anxiety and depression scores					
		HADS-A		P*	HADS-D		P*
		Above cutoff n (%)	Below cutoff n (%)		Above cutoff n (%)	Below cutoff n (%)	
e-AMD (n = 200)		83 (41.5)	117 (58.5)	< 0.001	127 (63.5)	73 (36.5)	< 0.001
Control (n = 120)		15 (12.5)	105 (87.5)		33 (27.5)	87 (72.5)	
Demographic characteristics (e-AMD)							
Income status	High-income group	5 (19.2)	21 (80.8)	0.004^a^	11 (42.3)	15 (57.7)	0.007^a^
	Middle-income group	55 (50.9)	53 (49.1)	0.047^b^	76 (70.4)	32 (29.6)	< 0.01^b^
	Low-income group	23 (35.4)	42 (64.6)	0.13^c^	26 (40.0)	39 (60.0)	0.84^c^
Gender	Female	48 (43.2)	63 (56.8)	0.57	76 (68.5)	35 (31.5)	0.10
	Male	35 (39.3)	54 (60.7)		51 (57.3)	38 (42.7)	
Smoking	Smoker	13 (33.3)	26 (66.7)	0.25	20 (51.0)	19 (49.0)	0.08
	Non-smoker	70 (43.5)	91 (56.5)		107 (66.5)	54 (33.5)	
Laterality	Unilateral	20 (32.3)	42 (67.7)	0.07	35 (56.5)	27 (43.5)	0.16
	Bilateral	63 (45.7)	75 (54.3)		92 (66.7)	46 (33.3)	
BCVA	< 20/40	45 (51.7)	42 (48.3)	0.01	58 (66.7)	29 (33.3)	0.41
	≥ 20/40	38 (33.6)	75 (66.4)		69 (61.1)	44 (38.9)	

*Chi-square test; n = sample size; ^a^comparison between high and middle-income groups; ^b^comparison between middle and low-income groups; ^c^comparison between high and low-income groups; HADS-A = anxiety scale; HADS-D = depression scale; BCVA = best corrected visual acuity in the better-seeing eye.

The patients with e-AMD had significantly lower mean scores than those of the controls for each of the items of the SF-36 QoL scale (P < 0.001). The test scores showed decreases with increasing age in both of the groups. The test scores declined with reduced vision, particularly in the domains of physical functioning and vitality, among the patients with e-AMD. The test scores also declined with extended disease duration (P < 0.001). The physical functioning and vitality scores were significantly higher among females than among males (P < 0.001) in the e-AMD group, but not in the control group. The data from the SF-36 scale in bilateral and unilateral e-AMD cases and in the control group are shown in Table 5.

**Table 5.** Comparisons of SF-36 quality-of-life scale between exudative age-related macular degeneration group and control group, and between unilateral and bilateral cases

SF-36 subscales	Exudative age-related macular degeneration				Control (n = 120)	P^b^
	All cases (n = 200)	Unilateral cases (n = 62)	Bilateral cases (n = 138)	P^a^		
Physical functioning	50.91 ± 25.9	56.2 ± 23.2	48.6 ± 26.9	0.05	64.9 ± 26.5	< 0.001
Role limitation due to physical health	13.12 ± 18.4	16.7 ± 19.9	11.5 ± 17.5	0.04	24.9 ± 22.2	< 0.001
Role limitation due to emotional problems	15.66 ± 20.0	18.8 ± 21.2	14.3 ± 19.3	0.13	29.7 ± 23.0	< 0.001
Energy/fatigue	47.10 ± 20.6	49.5 ± 19.6	46.0 ± 20.9	0.19	57.5 ± 15.7	< 0.001
Emotional wellbeing	51.66 ± 18.8	52.8 ± 18.1	51.1 ± 19.1	0.42	61.6 ± 14.2	< 0.001
Social functioning	53.68 ± 24.0	57.6 ± 22.7	51.9 ± 24.4	0.12	68.8 ± 20.6	< 0.001
Pain	54.18 ± 23.9	60.4 ± 22.3	51.3 ± 24.2	0.00	67.8 ± 21.7	< 0.001
General health	40.66 ± 19.7	44.8 ± 17.4	38.7 ± 20.4	0.01	53.5 ± 16.7	< 0.001

P^a^ = comparison of unilateral and bilateral cases; P^b^ = comparison of exudative age-related macular degeneration group and control group.

## DISCUSSION

The results from our study showed that patients with e-AMD were more likely to experience depression and anxiety symptoms and had lower QoL than did age-matched people with normal vision. These results have important personal and social implications. Older patients with impaired vision experience difficulties and anxiety in maintaining an independent lifestyle, since greater effort is required for participation in everyday activities, which leads to fatigue and limited mobility.

Previous studies have shown that there is a significant association between chronic eye diseases and depression. Depressionmay develop either as a result of a chronic physical disorder or due to a limitation on daily activities following vision impairment.^8^ Watsonetal. reported that anxiety often accompanies depression,^9^ and Mathew etal. found that 44% of the patients with AMD and 18% of the controls had depressive symptoms.^10^ Although reduced visual acuity is used as the primary outcome measurement in the majority of trials, loss of contrast sensitivity and color vision and increased susceptibility to glare from light are also associated with decreased vision-specific QoL among patients with AMD.^11^

A series of cohort studies conducted by Casten etal. showed that the prevalence of depression among patients with AMD was between 20% and 43%.^12^^,^^13^ Several researchers have assessed the relationship between severity of AMD and depressive symptoms.^14^^,^^15^ One of these studies suggested that there was no relationship between AMD and anxiety.^15^ A cross-sectional study found that the rate of depressive symptoms was not significantly different between patients with early AMD and those with late AMD (15.7% versus 17.2%).^16^ However, some other studies reported that the rate of depressive symptoms increased with the severity of e-AMD.^17^

In a cross-sectional study, the proportion of patients with e-AMD who presented depression was 17.9%.^27^ In case-control studies, the percentage of depression is usually found to be significantly higher among patients with e-AMD.^17^^,^^18^ Popescu etal. examined whether the percentage of depression among patients with AMD, glaucoma and Fuchs corneal dystrophy was higher than among controls and found that while it was 8% among the controls, it was 29% among patients with glaucoma and 39% among patients with AMD.^19^ In a controlled study conducted on patients with glaucoma and AMD, Kocak etal. showed that the percentage of depression was significantly higher among the patients with glaucoma or AMD than among the controls.^20^ However, the difference between the patients with glaucoma and those with AMD was not significant.

With regard to the relationship between AMD and anxiety, while several case-control studies failed to show any significant relationship,^18^ several others showed higher rates of anxiety among patients with AMD.^17^ Some studies did not find any relationship between the severity of AMD and anxiety symptoms.^17^^,^^18^ In the current study, we found significant differences in both the HADS-A and the HADS-D score between the patients with e-AMD and the controls.

The NEI-VFQ-25 test is among the most commonly used vision-specific QoL scales. Orr etal. found that NEI-VFQ-25 scores were positively correlated with good vision.^21^ In the Age-Related Eye Disease Study, the researchers assessed NEI-VFQ-25 scores twice, with a four-year interval between assessments, and demonstrated that there was a significant association between NEI-VFQ-25 scores and the progression of AMD and vision loss.^22^

A clinical study conducted on patients with e-AMD who received pegaptanib, ranibizumab or aflibercept showed that these treatments improved their QoL and reduced their dependence in relation to daily activities. In addition, it showed that these treatments decreased the incidences of depression and fall-related injuries.^23^ In the EQUADE study, using the NEI-VFQ-25 test, the researchers demonstrated that poor QoL was associated with impaired visual functions, prolonged duration of illness and poor social support (paid caregivers and home healthcare services).^24^ Ina study conducted on patients with e-AMD who received photodynamic treatment, their QoL was assessed using pre and post-treatment NEI-VFQ-25 test scores. Although visual acuity was maintained in 71% of the patients, photodynamic treatment failed to improve their QoL. In the current study, we found a significant correlation between improved visual acuity and increased NEI-VFQ-25 test scores. Poor visual acuity and prolonged treatment duration were associated with lower test scores and poorer QoL.

The SF-36 test is a practical instrument for assessing QoL among older adults. This test has, particularly, been used among patients with glaucoma. In a study performed in Turkey, using the SF-36 scale, it was shown that QoL was lower among patients with glaucoma.^25^ In another study, physical functioning, role-physical, bodily pain, social functioning and mental health scores were found to be significantly lower among the patients with glaucoma than among the controls.^26^ In a study in which the participants were divided into three groups, comprising patients with glaucoma, patients with a suspicion of glaucoma and controls, the patients with glaucomahad the lowest SF-36 scores and those with a suspicion of glaucoma hadthe second lowest scores.^27^ In the current study, the patients with e-AMD tended to score lower than the controls across all of the QoL domains. The test scores particularly showed that there were declines in the domains of physical functioning and vitality with increasing disease duration and reduced visual acuity.

Exudative AMD is a chronic disease that gradually causes significant central visual loss. Geographical, population and sociodemographic differences may lead to variable results regarding the scores on depression, anxiety and quality-of-life scales. These results can also be influenced by factors relating to patients’ expectations. Patients’ beliefs and fears relating to the disease and the ways in which they react to the disease need to be assessed. Compliance with treatment, in relation to chronic diseases, affects the course of the disease and the success of the treatment.

It should be borne in mind that psychiatric support may be needed in order to provide optimal compliance with treatment, in relation to mental health status and quality of life among e-AMD patients. The HADS-A, HADS-D, SF-36 and NEI-VFQ-25 scales can be used to fully ascertain the physical and mental health status of patients with e-AMD. Our study showed how our patient population with e-AMD was affected overall through undergoing treatment for chronic anti-VEGF treatment.

Compared with previous studies, the differences in the geographical, educational and sociodemographic characteristics of the participants in this study may, in part, account for the differences in the anxiety and depression rates and in the vision-specific QoL scores. Overall, we found that patients with e-AMD were especially at risk of impaired QoL. Therefore, these patients require close follow-up.

The limitations of this study include the small size of the sample that was used to investigate the outcome variables. The small population size may have limited the power of the study for making statistical differentiation to ascertain any slight significances in the subgroup analyses. In addition, the possible influence of the number of intravitreal anti-VEGF treatments for e-AMD on the quality-of-life scales was not evaluated. It would be valuable to study the different stages of the course of anti-VEGF treatment for e-AMD, such as the initial, midterm or long-term stage.

On the other hand, the use of three different scales to assess possible impairment of quality of life and emotional mood in the same population suffering from e-AMD was the strength of our study. Moreover, all the scales had also been validated in our country.

## CONCLUSION

Our results showed that patients with e-AMD had higher levels of symptoms of depression and anxiety and lower QoL, in comparison with the controls. The quality of life of these patients with e-AMD decreased as the disease duration increased and their visual acuity decreased. Our study indicated that treatments for patients with e-AMD should be managed in terms of dealing with their overall health. Further multicenter studies with large sample sizes are required to provide better validated data.
